# Physical Characteristics, Mineral Content, and Antioxidant and Antibacterial Activities of *Punica granatum* or *Citrus sinensis* Peel Extracts and Their Applications to Improve Cake Quality

**DOI:** 10.3390/plants11131740

**Published:** 2022-06-30

**Authors:** Hossam S. El-Beltagi, Nareman S. Eshak, Heba I. Mohamed, Eslam S. A. Bendary, Amal W. Danial

**Affiliations:** 1 Agricultural Biotechnology Department, College of Agricultural and Food Science, King Faisal University, P.O. Box 400, Al-Ahsa 31982, Saudi Arabia; 2 Biochemistry Department, Faculty of Agriculture, Cairo University, Giza 12613, Egypt; 3 Home Economics Department, Faculty of Specific Education, Assiut University, Assiut 71516, Egypt; nariman_saeed@aun.edu.eg; 4 Biological and Geological Sciences Department, Faculty of Education, Ain Shams University, Cairo 11341, Egypt; 5 Biochemistry Department, Faculty of Agriculture, Ain Shams University, Cairo 11241, Egypt; esslam136@gmail.com; 6 Botany and Microbiology Department, Faculty of Science, Assiut University, Assiut 71516, Egypt

**Keywords:** DPPH, ethanolic extract, polyphenolic, GC-MS, methanolic extract, yield

## Abstract

One-third of all food produced for human use is discarded as waste, resulting in environmental pollution and impaired food security. Fruit peels have bioactive compounds that may be used as antimicrobials and antioxidants, and the use of fruit peels is considered an alternative way to reduce environmental problems and agro-industrial waste. The aim of this study was to evaluate the phytochemical, mineral, extraction yield, total phenolic, total flavonoids, antioxidant, and antibacterial activity of several peel fruits, including *Citrus sinensis* (orange) and *Punica granatum* (pomegranate). The results revealed that pomegranate peel powder contains the highest amounts of ash, fiber, total carbohydrates, Ca, Fe, Mg, and Cu, while orange peel contains the highest amounts of moisture, protein, crude fat, P, and K. Furthermore, the aqueous and methanolic pomegranate peel extracts yielded higher total phenolic and total flavonoids than the orange peel extract. The identification and quantification of polyphenol compounds belonging to different classes, such as tannins, phenolic acids, and flavonoids in pomegranate peel and flavonoid compounds in orange peel were performed using UPLC-MS/MS. In addition, GC-MS analysis of orange peel essential oil discovered that the predominant compound is D-Limonene (95.7%). The aqueous and methanolic extracts of pomegranate peel were proven to be efficient against both gram-positive and gram-negative bacteria linked to human infections. Sponge cake substituting wheat flour with 3% pomegranate peel and 10% orange peel powder had the highest total phenolic, flavonoid compounds, and antioxidant activity as compared to the control cake. Our results concluded that pomegranate and orange peel flour can be used in cake preparation and natural food preservers.

## 1. Introduction

Foodborne infections are the main cause of illness or mortality, particularly in low-income nations due to inadequate sanitation or healthcare facilities. Many food-borne bacteria, such as enterohemorrhagic *Escherichia coli*, cause serious gastrointestinal diseases, including hemorrhagic diarrhea. The majority of such foodborne diseases are caused by pathogenic bacteria, viruses, or parasites and are a source of concern for worldwide public health [1]. Foodborne infections afflict around 600 million people worldwide each year, with approximately 420,000 people dying as a result of these illnesses [1]. The most typical symptoms of pathogen-induced foodborne infections are abdominal pain, diarrhea, fever, vomiting, or chills, which can lead to life-threatening dehydration or hemolytic uremic syndrome (HUS) [2]. *Salmonella enterica* is a gram-negative bacterial pathogen capable of infecting humans and animals and causing significant mortality worldwide [3]. Salmonella can survive undetected in the intestines of food animals and be transmitted to humans. Microorganisms such as *Salmonella* spp. and *S. enterica serovar typhimurium* can be influenced by animal diets and are considered clinically relevant intracellular bacterial agents that cause food poisoning or gastroenteritis in millions of people throughout the world every year [4]. There are requirements for the use of alternative control strategies, such as plant extracts and herbal products, particularly in developing countries due to the unavailability of antibiotic drugs that may cause harm to human health [2]. Plants/plant-derived compounds hinder bacterial growth through a variety of mechanisms. This may include preventing the bacteria from adhering to host cells [5], resulting in a decrease in microbe osmoregulation and a decrease in the transmembrane electrochemical gradient, boosting nitric oxide generation and producing a lethal effect [6], and the suppression of pathogen cell wall, protein, and nucleic acid formation [7].

Fruit wastes are produced in significant quantities throughout industrial processing, and their accumulation causes serious damage to the environment. Therefore, it must be managed or utilized. Fruit or vegetable waste products like peels, seeds, or stones can be successfully employed as a source of phytochemicals, antioxidants, or antimicrobials [8,9,10,11,12,13,14]. The majority of fruit waste had some antibacterial action and minor antifungal or yeast activity [15]. The fruit peels are high in minerals or phytochemicals and can be used as pharmaceuticals or as food additives [16]. Because they are important products, or their recovery may be economically beneficial, the innovative issues surrounding the utilization of these wastes as byproducts for more exploitation in the creation of high nutritional value food additives or supplements have garnered increasing interest [17]. The by-products are a good source of minerals, organic acids, sugars, dietary fiber, or phenolics, which have antibacterial, cardioprotective, antiviral, or anti-mutagenic properties [18].

There has recently been significant interest in using plant materials as an alternative method for controlling pathogenic microorganisms, and it has been shown that several plant products specifically target resistant pathogenic bacteria [19,20]. *Punica granatum* L. (Pomegranate) is one of the oldest fruits in the Punicaceae family (now in the Lythraceae family) [21]. Usually, pomegranates generate 669 kg of waste materials for every one ton of fresh fruit, with 78% composed of peel and 22% of seed [22]. Different studies report pomegranate peel as an interesting by-product [23]. Pomegranates are high in natural flavonoids and polyphenols, or proanthocyanidins, and they are widely eaten as nutritional foods for therapeutic purposes, health promotion, or antioxidant activity [24]. Pomegranate seeds were shown to scavenge reactive oxygen species (ROS) due to their high levels of vitamin K, folic acid, vitamin C, and polyphenols. Moreover, the pomegranate fruit pericarp includes a variety of bioactive substances, including gallic acid, ellagitannins, punicalagin, anthocyanins delphinidin, ellagic acid, pelargonidin, or luteolin [25,26,27], while pomegranate peel contains 25–28% polyphenols containing gallic acid and tannins for ROS scavenging [28]. These chemicals have the potential to be the most potent antibacterial agents in pomegranates [29].

Several studies have indicated that fruit peels, such as pomegranate peels, have antimicrobial properties [30]. Methanolic extracts of fruit, particularly the peel, have the strongest antibacterial effect [31]. The main components of pomegranate fruit are polyphenols, which show antibacterial activity via *Klebsiella pneumonia*, *Escherichia coli*, and *Pseudomonas aeruginosa* [32]. However, pomegranate peels have received less attention than natural preservatives in meat [33]. They also show improvement in food preservation by preventing harmful microorganisms that can cause food poisoning. Several studies were conducted to assess the efficacy of pomegranate isolate in sterilizing meat surfaces and maintaining food quality [33,34].

Citrus fruits, which are members of the Rutaceae family, are the most widely farmed fruits in the world. Citrus juice production generates waste that accounts for nearly half of the fresh fruit mass. This waste includes peels (50–55%), seeds (20–40% of fruit mass), pomace, and wastewater. Citrus waste includes ruined fruit, seeds, pulp, and peels. Every year, roughly 10 million metric tons of garbage are generated from the processing of citrus fruits worldwide, causing environmental problems [35]. Citrus peels include roughly 80% water, quickly attracting microorganisms, insects and mold or producing mycotoxins. As a result, the use of citrus peels may be required for waste management as well as income generation [36]. Citrus processing by-products have high amounts of natural flavonoids and phenolics [37,38], which are used as antimicrobials and antioxidants [39] and can also be used as a substrate for bacteria in the fermentation process for fuel production [40,41].

Bakery products, particularly cookies, are regarded as one of the most sustainable and satisfactory supplement carriers [42]. Although wheat flour is a good source of carbohydrates, it may lack significant enough concentrations of fiber, minerals, and biomolecules such as antioxidants to serve the increasing nutritional demands of vulnerable populations. Though adding flour to cookies raises concerns about consumer acceptability for color, taste, texture, and other baking properties [43], the product also has the potential to meet the nutritional requirements of the body. Furthermore, spongy cakes are the most popular bakery product due to their uniqueness, and they are frequently used in celebrations [44]; it grows by 1.5% per year worldwide [45]. It is typically made from wheat flour that has been extra-extracted due to a lack of crude fibers and phytochemicals. At the moment, crude fibers from alternative sources are available, such as vegetable, fruit, and their residues, which may provide crude fibers and bioactive compounds as natural components [46]. Many epidemiological studies confirm that crude fibers in fruit peel utilization help to prevent or reduce some cancer tumor types, obesity, and cardiovascular diseases [47]. Therefore, the fiber consumption is recommended to be up to 20 to 35 g daily [48].

The aim of this study was to verify antimicrobial or antioxidant characteristics for *C. sinensis* (orange) and *P. granatum* (pomegranate) peel extracts (ethanolic, methanolic, and distilled water) against *P. aeruginosa*, *S. macescens*, *E. coli*, *B. subtilis*, *B. cereus*, *S. aureus*, or *K. pneumoniae* and perform an analysis of their phytochemicals like phenols and flavonoids, which are responsible for antimicrobial activity. Furthermore, the current study was designed to investigate the nutritional potential of pomegranate and orange peel powder by incorporating it into wheat flour to produce acceptable and nutritionally enriched cakes, as well as to determine some of their physical and sensory properties.

## 2. Materials and Methods

### 2.1. Fruits

Fresh *C. sinensis* and *P. granatum* were purchased from the supermarket in Assiut city, Assiut, Egypt. The selected *P. granatum* or *C. sinensis* fruits were ripe or had no signs of injury or infection. Fruits were washed in tap water and were left to dry at room temperature for one hour. The experiments were generally performed immediately after purchase.

### 2.2. Preparation of P. granatum and C. sinensis L. Peel Powders

The fruits were sanitized with 70% alcohol before being washed with sterile distilled water and peeled. Peels were chopped into pieces and dried overnight in a hot air oven at 50 °C, then processed to a fine powder in a laboratory mill. The powder was placed into plastic dark bags and kept at room temperature until it was utilized.

### 2.3. Physical Analysis of P. granatum and C. sinensis L. Peel Powders

The *P. granatum* and *C. sinensis* L. peel powders were analyzed through sensory evaluation. Every peel was assessed by test in terms of color, odor, and appearance. These parameters were noticed visually.

### 2.4. Chemical Composition of P. granatum and C. sinensis L. Peel Powders

The AOAC [49] was used to determine the crude fiber content of *P. granatum* and *C. sinensis* L. peel powders. Crude fat in fruit peels was determined using a soxhlet extractor unit [50]. Total carbohydrates were determined using a spectrophotometer in accordance with the AOAC [51]. Nitrogen was determined using a kjeldahl procedure according to the AOAC [49]. Each experiment was carried out in triplicate.

### 2.5. Mineral Content of Fruit Peel Powders

To achieve a constant weight, one gram of each sample was passed through porcelain crucibles that were turned on in the muffle at 550 °C. Ash has been solubilized in 3 mL of 3 M hydrochloric acid and then completed to 100 mL [51]. Different minerals such as Ca, Fe, Mg, and Cu in *P. granatum* and *C. sinensis* peel powders were determined by ICP6200 (Inductively Coupled Plasma Emission Spectrometer). Phosphorus was determined using a spectrophotometer and potassium was determined using the flam photometer as described [51]. All experiments were conducted in triplicate.

### 2.6. Preparation of Extracts

For two weeks, a 20 g powder of each peel sample was mixed into different solvents, including methanol, ethanol, or water. The extraction process was used in the conical flask, where the flask was closed with cotton wool and wrapped with aluminum foil. The fruit extracts were then centrifuged at 10,000× *rpm* for 15 min at 40 °C [52]. The homogeneous mixtures were filtered and used to evaluate antibacterial, antioxidant, or phytochemical activities. To prepare samples, 20 g of ground peel were separately soaked in 100 mL solvents. The extract was prepared in three types of solvents, i.e., ethanol, methanol, and water. The samples were incubated at 37 °C for 24 h in a shaking incubator with 200 rpm. The fruit extracts were then centrifuged at 10,000× *rpm* for 15 min at 40 °C. Then the samples were filtered with Whatman no. 1 filter paper and the filtrate was stored in the incubator at 4 °C. This extraction procedure was repeated three times to extract maximal components from the peel. The pooled extract was used for the analysis of phenolics, antioxidant, and antibacterial activities [53].

### 2.7. Phytochemical Screening of Peel Extracts

The phytochemical screening of ethanol, methanol and dist. water extracts was carried out in accordance with Trease and Evans [54]. Alkaloid, tannins, saponin, cardiac glycosides, flavonoids, glycosides, phenols, saponins, steroids, and terpenoids were among the phytochemicals examined.

### 2.8. Total Phenolic Content

The Folin-Ciocalteu reagent procedure [55] was used to measure the phenol concentration in peel extracts with slight modifications. The reaction mixture included extract (100 µL), Folin-Ciocalteu reagent (100 µL), and 20% sodium carbonate (3 mL). After 1 h of incubation at room temperature, the absorbance of the dark blue complex was taken at 765 nm. Gallic acid has been utilized as a standard with concentrations ranging from 200 to 1000 ppm. Total phenolic content was estimated as mg of gallic acid equivalents per 100 g of dry sample weight.

### 2.9. Total Flavonoid Content

The total flavonoid content of extracts was estimated using the procedure reported [56]. The reaction mixture of extracts (500 µL), distilled water (2 mL), or 5% NaNO_2_ (0.15 mL) was incubated at room temperature for 6 min before serving 10% AlCl_3_ (0.15 mL) solution, 2 mL of 4% NaOH solution, and the complete mixture to a final volume of 10 mL by water. The absorbance of the reaction mixture was taken at 510 nm. The calibration curve uses quercetin as a reference. Total flavonoid content was calculated as mg of quercetin equivalents (QE) per gram of dry sample weight.

### 2.10. Extraction of Polyphenolics from Orange and Pomegranate

About 10 g of fresh grated peels of both orange and pomegranate were extracted by soaking them in 50 mL of methanol 99.9% Sigma HPLC grade overnight at 25 °C in a dark bottle. The solvent was filtered (Watman No.1) and the solids were re-soaked in 50 mL of fresh methanol overnight, and then the filtrate pooled. The filtrate was concentrated under reduced pressure at 45 °C and 2 mL of the extract was filtered through 0.22 µm PFTE and kept in a 1.5 mL amber HPLC vial at −20 °C until analysis.

### 2.11. UPLC-MS Analysis Conditions

LC-MS/MS (liquid chromatography-tandem mass spectrometry) analyses were performed as described by Abid et al. [57] on an Acquity UPLC-XEVO TQD, Waters System, USA consisting of an Accela U-HPLC unit with a photodiode array detector and an LTQ Orbitrap XL mass spectrometer fitted with an electrospray source. Chromatography was performed on 5 μL sample injections onto a C18 column (Acquity UPLCBEH C18, 1.7 mm, Waters, Milford, MA, USA) using a 400 µL/min linear mobile phase gradient of methanol/acetonitrile 50:50 (solvent A) and water 1% formic acid (changing from 5%, A: 90 A over 60 min, followed by an isocratic phase for 5 min and then a column wash phase and equilibrium of the column for 3 min before the next injection). The electrospray ionization (ESI) source of the mass spectrometer was operated in negative and positive mode using the collision-induced dissociation (CID) to protonate and deprotonate fragments; the positive and negative mode were compared since the phenolic compounds in question ionize better in negative mode. The orbitrap mass analyzer was set to scan in the range of *m*/*z* 200–2000 at 30,000 resolutions in positive and negative polarity, while the linear ion-trap analyzer performed MSn analyses on the most abundant ions in both polarities using an ion isolation window of ±2 *m*/*z* and a relative collision energy of 35%.

### 2.12. GC-MS Qualitative Identification

The essential oil fraction was extracted from fresh grated orange peels (about 25 g) by petroleum ether and the solvent was removed. The separation of the components of the orange essential oil has been carried out in 1 µL of sample solution (100 μg extract/mL) in hexane: diethyl ether 1:1 ratio by the GC-MS (Shimadzu-QP-2010S plus) instrument equipped with [AOC-20i+s] autosampler autoinjector and a capillary column (Rtx-1 30 m × 0.32 mm I.D., 0.25 μm). The oven temperature was adjusted for an initial temperature of 50 °C followed by a 5 °C/min temperature ramp to 180 °C and held for 1 min, then raised to 250 °C by 10 °C/min. The final temperature was maintained for 1 min. Injector and mass interface temperatures are adjusted at 250 °C. Helium carrier gas (He) column flow was 2.62 mL/min with a linear velocity of 58.7 cm/s. The mass parameters were set as follows: ion source temp. of 210 °C, solvent cut time of 4.0 min, MS detector (EI-mode) start time of 4.1 min, and end time of 34.4 min; the compounds were acquired by scan mode ACQ start of *m*/*z* 70 and end of *m*/*z* 500. The integration was performed by Lab Solution software 4.1 for GC solution Ver.2.5 (Shimadzu, Tokyo, Japan) and compounds were compared by NIST -NIH EI-MS LIBRARY 2020 (National Institute of Standard and Technology) (Shimadzu, Tokyo, Japan).

### 2.13. DPPH Assay

The antioxidant activity of each peel was assessed by examining the effect of each peel extract on DPPH scavenging. Five hundred µL of each peel extract was thoroughly shaken or mixed with 3 mL of a 0.002% methanolic DPPH solution. After 30 min in the dark, the absorbance was read at 517 nm using a spectrophotometer for each sample solution or blank (containing only DPPH) [58]. Every measurement was taken in triplicate. The reference materials were ascorbic acid and butylated hydroxyl toluene (BHT) in various concentrations. The peel’s scavenging potential was calculated in terms of DPPH inhibition percentage (%) utilizing following the formula:

$$\%\mathrm{DPPH}\;=\;\frac{A\;\mathrm{blank}\;-\;A\;\mathrm{sample}}{A\;\mathrm{blank}}\;\times \;100$$

where A blank represents the absorbance of a blank sample and A sample represents the absorbance of an extracted fruit.

### 2.14. Scavenging of Hydrogen Peroxide

Extracts’ ability to scavenge hydrogen peroxide has been estimated by utilizing standard procedures [59]. A hydrogen peroxide solution (40 mM) was prepared in a phosphate buffer (pH 7.4). The peel extract was mixed into 0.6 mL of hydrogen peroxide solution. After 10 min, hydrogen peroxide absorption was measured at 230 nm in comparison to a blank solution containing a phosphate buffer but no hydrogen peroxide. The reference materials were ascorbic acid and butylated hydroxyl toluene (BHT) in various concentrations. The proportion of hydrogen peroxide scavenged by peel extracts was estimated as follows:

$$\%\;\mathrm{Scavenged}\;H_{2}O_{2}\;=\;\frac{Ao\;-\;A1}{Ao}\;\times \;100$$

where Ao was the absorbance of control and A1 was the absorption in the existence of a sample of extract.

### 2.15. Determination of Extraction Yield

All extracts were dried for 12 h in a hot air oven set at 105 °C. Extract yields were determined as a percentage of dry weight. The percentage yield of extracts was estimated by dividing the weight of the starting plant material by the weight of the extracts. The yield is given as a percentage (%) and computed as follows:

$$Y\;=\;\frac{We\;}{Wp}\;\times \;100$$

where “*Y*” is extraction yield (%), “We” is weight of extract (g), and “Wp” is weight of peel (g).

### 2.16. Isolation and Identification of Bacterial Pathogens

Bacterial pathogens were isolated from human infected samples such as urine or pus at the Assiut University Hospital and their identification was carried out morphologically and genetically. Nutrient agar, blood agar, Eosin methylene agar, and MacConkey agar media were utilized in the isolation or identification of bacterial isolates.

### 2.17. Morphological or Biochemical Test

Morphological evaluation was performed using a light microscope. Biochemical or physiological analyses were performed in accordance with procedures outlined in Bergey’s Manual [60,61]. The phenotypes of isolates were investigated (motility, gram stain, and morphological or biochemical properties).

### 2.18. S rDNA Sequencing or Phylogenetic Analysis

The sample’s total genomic DNA was isolated or purified. For PCR amplification of the variable regions of 16S rDNA from purified genomic DNA, the primer sets F (5-AGA GTT TGA TCC TGG CTC AG-3) by GC clamp or R (5-GGT TAC CTT GTT ACG ACT T-3) at a 65 °C annealing temperature were utilized. The PCR product was then cleaned using the Gene JETTM PCR Purification Kit (Thermo, Waltham, MA, USA). The loading of 4 µL of PCR mixture was performed to analyze PCR results on 1% agarose gel via a 1 Kb plus ladder (Fermentas). Finally, PCR products were sequenced by the GATC company using ABI 3730xl DNA sequences and forward or reverse primers.

### 2.19. The Activity of Antibacterial Extract

The bactericidal activity of various extracts of *C. sinensis* and *P. granatum* peel was measured utilizing agar well disc diffusion methods, as previously reported [62]. Bacterial culture was carried out using nutrient agar media. Under sterile conditions, wells (5.0 mm in diameter) were cut from the agar and 1.0 g of lyophilized extract was dissolved in 6 mL of deionized water (1:6 *w*/*v*), and 10 L was directly added into the wells of agar plates. The inoculated plates were incubated at 37 °C for 48 h. At the end of the incubation period, inhibition zones formed on the medium and the diameter of the inhibition zone was measured and recorded as the mean diameter (mm).

Prior to every experiment, the optical density (OD) for bacterial growth (10^7^ CFU/mL) was determined using a spectrophotometer with a wavelength of 600 nm. Each experimental outcome was replicated three times. The extracts’ antibacterial activity was also assessed using standard antibiotics via the (AI) activity index.

### 2.20. Sensitivity Test of Antibiotics

The agar disc diffusion procedure was used to investigate the sensitivity of antibiotics, such as amoxicillin (10 g), against all tested bacterial strains [63,64].

### 2.21. Minimum Inhibitory Concentration (MIC)

To determine minimum inhibitory concentrations (MIC) of active extracts, the agar dilution method was used. At 37 °C, stationary phase cultures of the tested bacterial spp. were produced and utilized to inoculate a fresh 5.0 mL culture to an OD600 of 0.05. Cultures were cultured at 37 °C for 5.0 mL until an OD600 of 0.10 was reached, after which the standardized bacterial suspensions were made to a final cell density of 4 × 10^6^ CFU/mL. Serial dilutions of tested compound samples have been prepared or combined with 5.0 mL of standardized bacterial suspension, and then added to plates incubated for 24 h at 37 °C. Every dilution, colony-forming units (CFU) were recorded or compared to the growth of untreated controls. The smallest concentration of peel extracts capable of killing microorganisms was determined as the minimum inhibitory concentration (MIC).

### 2.22. Preparation of Cakes

Cakes were prepared using the standardized recipe and method given by Sharoba et al. [65] and modified by Zaker et al. [66]. The formula used was as follows: 250 g wheat flour, 125 g sugar, 53.50 g fat, 12.50 g of baking powder, 110 g fresh whole egg, 25 g dry milk, 2 g vanilla, and 70–72 mL water. The fat was mixed until the color became white, then sugar was added to butter and mixed until it got smooth like cream, and then a well-blended egg and vanilla were added and mixed together. Cakes were made from 100% wheat flour (control). The blend’s wheat flour (72%), with orange and pomegranate peel as antioxidant sources, were replaced with wheat flour at 10% levels of orange peel and 3% of pomegranate peel; the wheat flour and baking powder were stirred together and added alternately to the egg mixture. The mixture was whipped until smooth. The dough was transferred to a greased pan and baked for 25 min at 200 °C, then it was cooled at room temperature. Cakes were prepared according to the formula shown in Table 1.

### 2.23. Shelf Life Effect upon Sensory Evaluation of Cake Supplemented with C. sinensis and P. granatum Peel Powder at Room Temperature (30 °C) and Refrigeration Temperature (3 °C)

The sensory evaluation was done on a 9-point hedonic scale as per the method given by Zaker et al. [66]. A sensory evaluation of prepared cake was conducted by a 30-member trained panel comprised of postgraduate students and academic staff members from Assuit University’s Specific Education Faculty to determine the extent of consumer acceptance and its effect on cake shelf life. Judgments were made through the rating of products on a 9-point hedonic scale with corresponding descriptive terms ranging from 9 ‘like extremely’ to 1 ‘dislike extremely’. The shelf life according to these terms was investigated by storing the cake supplemented with *C. sinensis* and *P. granatum* peel powder at room temperature (30 °C) and refrigeration temperature (3 °C).

### 2.24. Analysis of the Microbial Load in Cake during the Storage Period

Microbial load was analyzed using the pour plate method according to Vanderzant and Splittstoesser [67]. The cake (1 g homogenized sample) was aseptically mixed in 9 mL of sterile saline solution (0.85% NaCl) and the samples were serially diluted before analysis. The appropriately diluted samples (1 mL) were poured into sterilized petri plates and warm (50 °C) nutrient media was poured, mixed, and allowed to solidify. The media was used for the evaluation of the total plate count and mold count during storage period. The colonies developed after incubation were counted and expressed as CFU/g.

### 2.25. Statistical Analysis

Statistical data analysis was performed utilizing a one-way ANOVA test (Analysis of variance) with SPSS software version 21.0 (Chicago, IL, USA). Duncan multiple range tests were calculated at 0.05 levels.

## 3. Results and Discussion

### 3.1. Physical Characteristics of Fruit Peel Powders

The results of the physical characteristics of *P. granatum* and *C. sinensis* powder are represented in Table 2. The pomegranate peel powder has a dark brown color and a strong tannic odor, while the orange powder has a light brown color and a pleasant odor. These results are in accordance with Kaur et al. [68], who reported that the dried peels of *P. granatum* L. are brittle and dark reddish brown in color with variations in size. In addition, Hanafy et al. [69] found that the highest amount of tannin content was reported for pomegranate extracts, followed by orange, and then banana. Due to the high amounts of tannins in pomegranate peel, the odor of pomegranate extracts is tannin.

### 3.2. Chemical Composition and Mineral Content of Fruit Peel Powders

**Figure 1:**
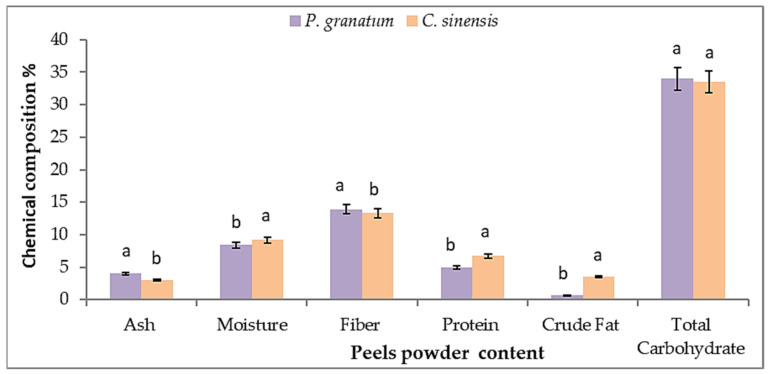
Chemical composition of *P. granatum* and *C. sinensis* peel powder (%). Each value is a mean (±SD) of three replicates. The different letters on the same bar show a significant difference according to Duncan’s test at *p* ≤ 0.05.

The chemical composition of *P. granatum* and *C. sinensis* peel *L.* powders is represented in Figure 1. Pomegranate peel powder contains the highest amount of ash (4.0 ± 0.2%), fiber (13.9 ± 0.2%), and total carbohydrates (33.97 ± 1.2) compared to orange peel, which contains less ash (3.0 ± 0.2%), fiber (13.3 ± 0.3%), and total carbohydrates (33.55 ± 1.4%). On the other hand, orange peel includes the highest quantity of moisture (9.18 ± 0.3%), protein (6.72 ± 0.6%), and crude fat (3.52 ± 0.5%) compared to pomegranate peel powder, which contains less amount of moisture (8.4 ± 0.3%), protein (4.98 ± 0.5%), and crude fat (0.68 ± 0.1%). These findings are consistent with those of Romelle et al. [70], who discovered that pomegranate peel powder has more ash (6.07 g/100 g DW), crude fiber (17.63 g/100 g DW), and total carbohydrates (59.89 g/100 g DW) than orange peel, which has less ash (5.17 g/100 g DW), crude fibers (14.19 g/100 g DW), and total carbohydrates (53.27 g/100 g DW). On the other hand, orange peel includes the highest quantity of protein (9.73 g/100 g DW) and crude lipids (8.7 g/100 g DW) compared to pomegranate peel powder, which contains less protein (3.46 g/100 g DW) and crude lipids (3.36 g/100 g DW).

The pomegranate fruit peel powder is considered a good source of crude fiber, ash, and carbohydrates. In this regard, the peel of pomegranate fruits can be used as a good source of bioactive compounds such as crude fibers which provide multiple health benefits, such as their ability to lower serum LDL cholesterol levels, enhance glucose resistance and insulin levels, decrease hyperlipidemia and hypertension, contribute to gastrointestinal health, and inhibit certain cancers such as colon cancer [71]. Nonetheless, the most pressing issue with orange peels is their high moisture content (75–90%), which makes them very perishable with a short storage life [72]. As a result, in order to maintain them for future use, their water levels have to fall.

The mineral composition of *P. granatum* and *C. sinensis* L. peel powders were evaluated and the obtained results are recorded in Table 3. Data showed that *P. granatum* L. peels contain higher concentrations of Ca, Mg, Fe, and Cu than *C. sinensis* peels. On the other hand, *C. sinensis* peels contain higher concentrations of P and K than *P. granatum* peels. These results are consistent with that of Abdel Wahab et al. [73], who showed that orange peel contains significant concentrations of potassium. In general, it can be stated that *P. granatum* peels are distinguished by their high concentration of nutritional minerals and are regarded as a good source of macro or micro elements.

These results are similar to those of Sroka and Cisowski [59]. Furthermore, the peels of *P. granatum* and *C. sinensis* are considered a source of minerals necessary for the normal functioning of the body system. The use of these peels will improve the recycling of waste and will also help with solid waste management and decrease environmental impact [74].

### 3.3. Phytochemical Screening

Alkaloids, tannins, flavonoids, or phenolic constitutes are the most important bioactive compounds derived from plants [75,76]. Our findings in Table 4 demonstrated that the peels of *C. sinensis* and *P. granatum* were a wealthy source of phytochemicals that were uncommon in other plants or became potent via a variety of pathogens. The aqueous and methanolic extract of each peel contains high amounts of qualitative phytochemicals compounds like alkaloids, tannins, flavonoids, glycosides, phenols, steroids, and teraponids. This could be owing to the strong polarity of methanolic solvent, which can extract a wider range of plant components than other solvents [77]. The *P. granatum* contain higher amounts of phytochemicals than *C. sinensis*. A lot of biological and therapeutic activities have been identified for these secondary metabolites [78]. Phytochemicals can defend your health in a variety of ways. Polyphenols, carotenoids, and other antioxidants help protect cells from free radical damage [79]. They may also assist in the reduction of cancer risk by blocking tumor development [80]. Other routes of action include antimicrobial activity and hormonal stimulation [81]. The variation in antibacterial activity when extracted to multiple solvents from the same source demonstrates that not every phytochemical responsible for antibacterial activity is dissolvable in a single solvent [82].

### 3.4. Total Phenolic and Flavonoid Content

Several studies have found that plant phenol content is connected to antioxidant activity, probably linked to their redox characteristics, which operate as reducing agents, hydrogen donors, or singlet oxygen quenchers [83,84].

The data in Table 5 showed that higher concentrations of total phenolic content were detected in water (513.8 ± 4.0 and 160.3 ± 3.0 mg gallic acid/100 g) and the methanolic extract (490.6 ± 4.0 and 155.4 ± 2.0 mg gallic acid/100 g) of *P. granatum* and *C. sinensis*, respectively, than in the ethanolic solvent. These results illustrate the effect of solvents on the extractability of phenols. The findings of this study indicate that the nature of the solvent has a significant impact on the ability of phenolic plants to extract phenol [85]. The total phenolic content (TPC) of *C. sinensis* and *P. granatum* derived with methanol was dramatically higher than that extracted by ethanol extract. The extracted TPC was higher and significantly increased in *P. granatum* compared to *C. sinensis*. These findings are consistent with those of Selahvarzi et al. [86] who discovered that the level of total phenolics in pomegranate peel extract (2.701 mg GAE/g extract) was substantially greater than in orange peel extract (1.861 mg GAE/g extract). Pomegranate peel extract (72.12%) had stronger antioxidant activity than orange peel extract (54.35%).

The variances could be due to the nature or the peculiarities of the peel types. The differences in TPC values for different peel types could be influenced by environmental factors, the degree of fruit ripening, or genetic factors [84]. Furthermore, the polarity of the solvent has a significant impact on enhancing the extract’s content [87].

Phenolic compounds’ antioxidant activity differs according to its molecular structure. The antioxidant activity of plant extracts is powerfully affected by the extract’s composition, the conditions under which it is tested, and the techniques utilized to assess its activity [86]. These bioactive compounds’ antioxidant activity is owing to their redox characteristics or chemical structures that could play essential roles in neutralizing free radicals, chelating heavy metals, or reactive oxygen spices [88].

In addition, the water and methanolic extracts of pomegranate peel contain higher concentrations of total flavonoids compared to the orange peel (Table 5). The concentrations of total flavonoids content were about 45.3 ± 0.5, 40.3 ± 0.3 mg QE/g in water and methanolic extract of the pomegranate peel, respectively, and about 22.2 ± 0.5, 15.7 ± 0.3 mg QE/g in water and methanolic extract of the orange peel, respectively. Flavonoids have significant antibacterial and antifungal properties [89].

### 3.5. UPLC-MS Analysis of Polyphenols

#### 3.5.1. UPLC-MS Analysis of Polyphenols in Orange Peel

**Figure 2:**
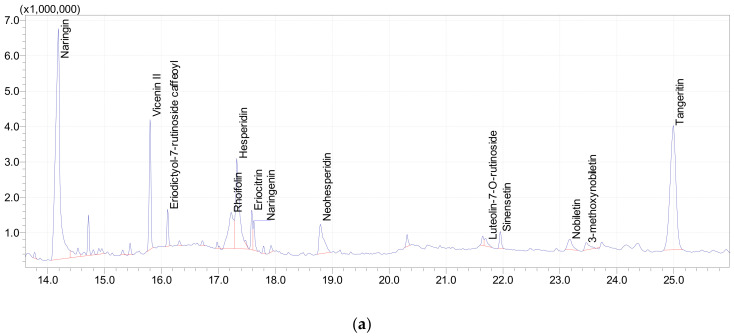

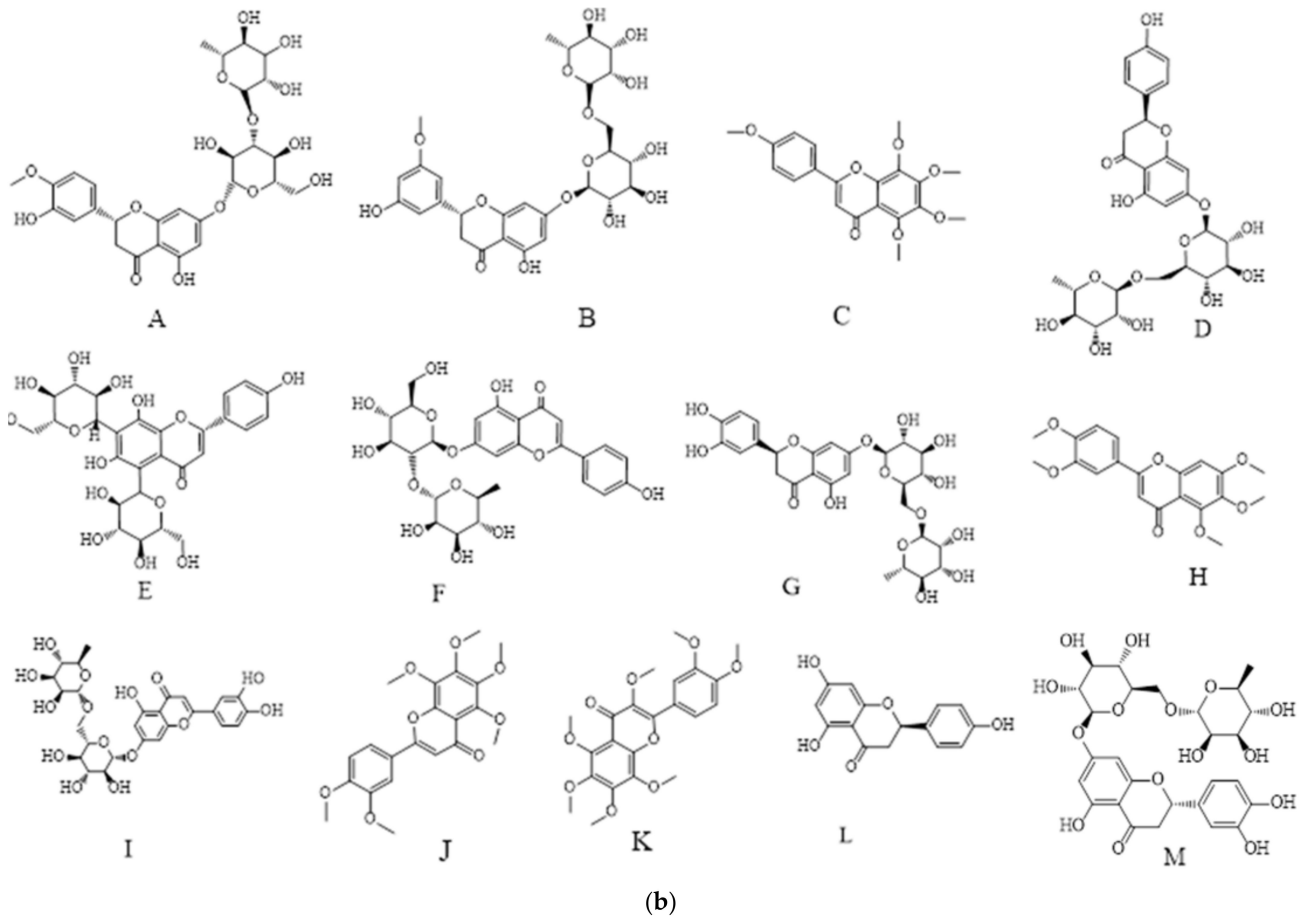
(**a**). Phenolic compounds of *C. sinensis* peel powders by LC-MS/MS. (**b**). Chemical structures of the major phenolic compounds in *C. sinensis* peel extract. A: Hesperidin; B: Neohesperidin; C: Tangeretin; D: Narnigin; E: Vicenin II; F: Apigenin 7-O-neohesperidoside. G: Eriocitrin. H: Sinensetin. I: Luteolin-7-O-rutinoside. J: Nobiletin K: 3-methoxynobiletin. L: Naringenin. and M: Eriodictyol-7-rutinoside.

An ultra-performance liquid chromatography combined with mass spectrometry (UPLC-ESI-MS/MS) was used to identify the phenolic compounds in the methanolic extract of orange peels. Table 6 and Figure 2a,b summarize the 12 compounds identified by UPLC-ESI-MS/MS and their characteristics including the retention time and percentage of each compound. The major polyphenolic compounds were naringin (31.75%), hesperidin (10.1%), vicenin II (6.61%), Apigenin 7-O-neohesperidoside (Rhoifolin) (5.91%), and neohesperidin (3.9%). The results indicated that orange peel is a rich natural source of several phenolic compounds that are well-known for their antioxidant and antimicrobial activities [90]. These results are in accordance with Shehata et al. [91] (2021) who found the major compounds were narirutin (~20%), naringin (~18.2%), hesperetin (~11.8%), datiscetin-3-O-rutinoside (11.5%), and sakuranetin (~6%). Compounds detected at low concentrations (~2–4%) include cynaroside A, isoorientin, flavanone base +3O, C-Hex, diosmetin-7-O-rutinoside, and didymin, and some compounds represented ~1% or less. Also, Safdar et al. [92] (2017) found that naringin is the predominant flavanone glycoside flavonoids in kinnow citrus peel.

#### 3.5.2. UPLC-MS Analysis of Polyphenols in Pomegranate Peel

**Figure 3:**
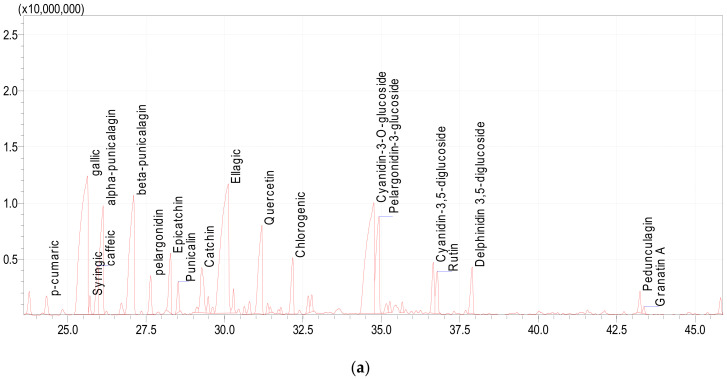

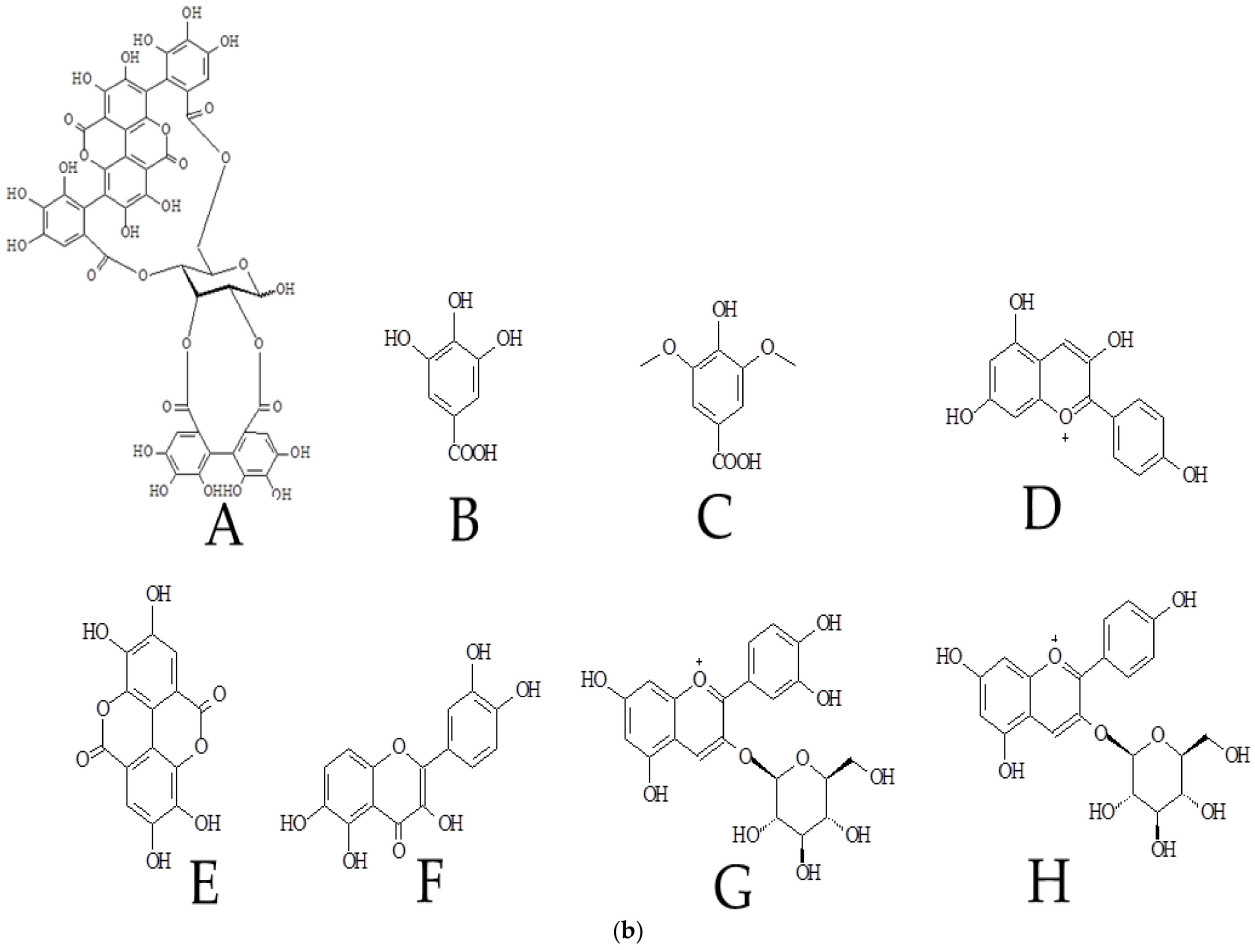
(**a**) Phenolic compounds of *P. granatum* peel powders by LC-MS/MS. (**b**) Chemical structure of the phenolic compounds in *P. granatum* peel extract. A: α- and β-Punicalagin; B: Gallic Acid; C: Syringic acid; D: Pelargonidin; E: Ellagic acid; F: Quercetin; G: Cyanidin-3-O-glucoside; H: Pelardonidin 3-O-glucoside.

Table 7 and Figure 3a,b summarize 20 compounds identified by UPLC-ESI-MS/MS and their characteristics including the retention time and percentage of each compound. The major polyphenolic compounds were gallic (17.07%), ellagic (16.54%), cyanidin-3-O-glucoside (14.54%), beta-punicalagin (9.25%), alpha-punicalagin (6.34%), quercetin (5.64%), and pelargonidin-3-glucoside (5.44%). The punicalagin in pomegranate peel extract represented the most dominant component in the extract compared with ellagic acid, gallic acid, catechin, and epicatechin [93]. Our results confirmed that the phenol composition of pomegranate is strongly influenced by the fruit parts (such as peel, mesocarp, and arils), cultivar, environmental conditions, solvent, and methods used for the extraction, as also reported in other studies [94].

### 3.6. Essential Oil Compounds in C. sinensis Peels by GC-MS

**Figure 4:**
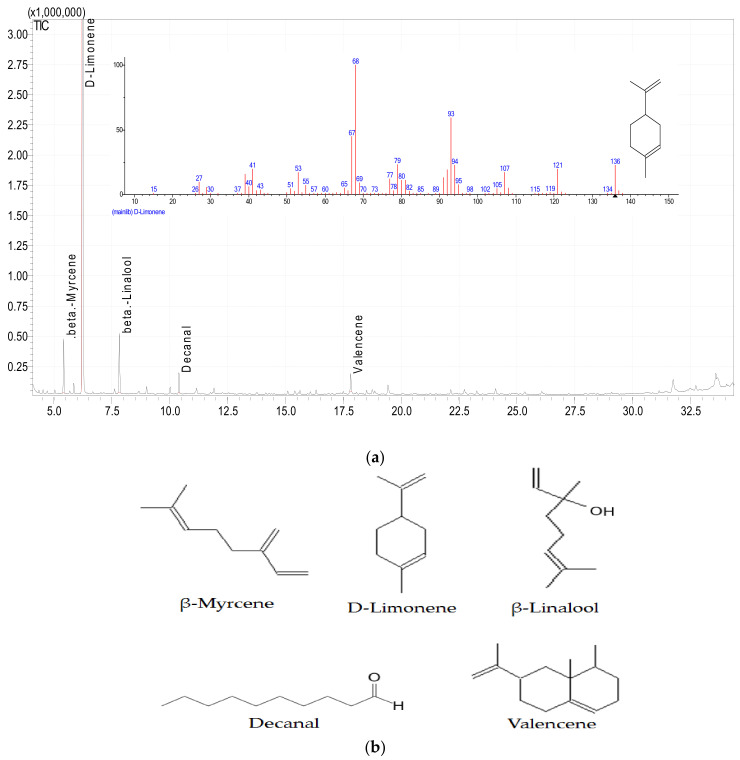
(**a**). Essential oil compounds of orange peel powders by GC-MS. (**b**). Chemical structure of the different compounds in the *C. sinensis* peel essential oil.

The GC-MS chromatogram of the orange peel oil extract of *C. sinensis* displayed five peaks, indicating the presence of five compounds. The chemical compounds identified in the methanolic peel oil extract of *C. sinensis* are presented in Table 8 and Figure 4a,b. GC-MS analysis discovered that the predominant compound is D-Limonene (95.7%). Citrus species peels normally contain more than 70% limonene [95]. In the present study, 65% of limonene was detected. The major component of the oil is D-limonene, which is probably the antibacterial and antifungal property of the oil [96]. Limonene is also highly useful in agriculture as it is an insect repellent [96].

### 3.7. Antioxidant Activity

#### 3.7.1. Radical Scavenging Activities (DPPH)

**Figure 5:**
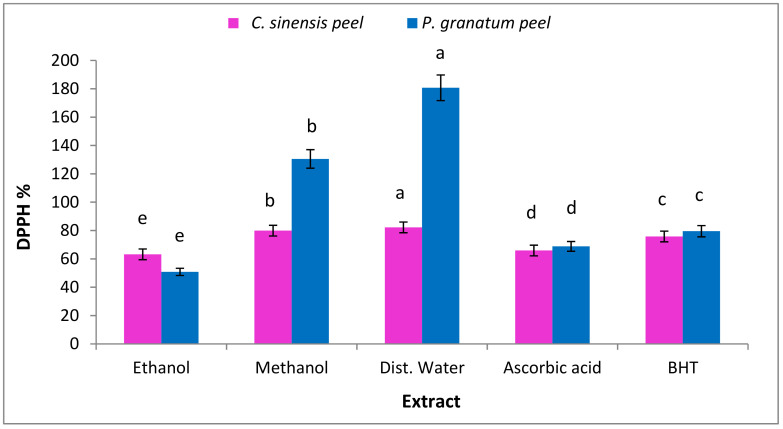
Antioxidant activities of *C. sinensis* and *P. granatum* peel extract against DPPH^•^. Each value is a mean (±SD) of three replicates. The different letters on the same bar show a significant difference according to Duncan’s test at *p* ≤ 0.05.

DPPH is a stable, free organic radical with an absorption band of roughly 515–528 nm that is often employed as a reagent to assess antioxidants’ free radical scavenging potential [97]. Data in Figure 5 indicated that antioxidant activity by DPPH was higher in the water (180.7 ± 4.0; 82.2 ± 0.2) and methanolic extract (130.5 ± 2.0, 79.9 ± 0.3) of *P. granatum* and *C. sinensis* peel, respectively. Foods high in antioxidant phytoconstitutes have been shown to be effective in preventing oxidative stress-related diseases, such as cancer and heart disease [98,99]. Antioxidant molecules, both enzymatic and non-enzymatic, operate as potent defensive mechanisms, interacting with hazardous substances to prevent their negative effects. Unfortunately, despite their high efficacy, these identical defenses have limited ability and can be overcome, resulting in a rise in ROS that can promote the development of dermatological illnesses [100]. Synthetic antioxidants (like butyl hydroxyanisole or butylhydroxytoluene) have been commonly utilized to control or protect against ROS-mediated diseases for decades. Nevertheless, given potential health concerns or toxicity associated with synthetic antioxidants, prevailing research is focusing on natural antioxidants as an alternate strategy for restoring homeostasis in the oxidant system [98].

#### 3.7.2. Hydrogen Peroxide Scavenging Activity

**Figure 6:**
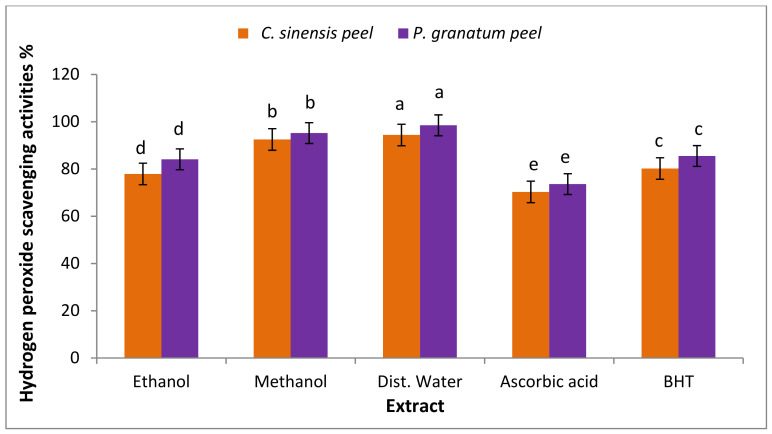
Hydrogen peroxide scavenging activity of *C. sinensis* and *P. granatum* peel extract. Each value is a mean (±SD) of three replicates. The different letters on the same bar show a significant difference according to Duncan’s test at *p* ≤ 0.05.

Hydrogen peroxide is a highly reactive oxygen species that can destroy a wide range of biological substrates, including carbohydrates, DNA, proteins, or polyunsaturated fatty acids. Preventing such hazardous interactions is critical for human health as well as the shelf life of foods, cosmetics, and medications [97]. As shown in Figure 6, *C. sinensis* and *P. granatum* peel scavenged hydrogen peroxide by utilizing a hydrogen peroxide procedure. The aqueous and methanolic extracts of orange peel (94.4 ± 0.5, 92.5 ± 0.5) were lower than pomegranate peel extracts (98.5 ± 2.0, 95.2 ± 2.0), respectively. Hydrogen peroxide is the most unstable or reactive, or it has a high oxidative capacity, swiftly interacting with practically every molecule in its proximity [101]. Like most reactive oxygen species, hydrogen peroxide can induce lots of biological effects like mutation, cell death, cancer, and aging [102]. The hydroxyl group of phenolic substances inhibits the generation of ROS, or free radical scavenging [103]. As a result, consuming foods that can scavenge hydrogen peroxide may help to reduce its negative effects [104].

### 3.8. Extraction Yield

**Figure 7:**
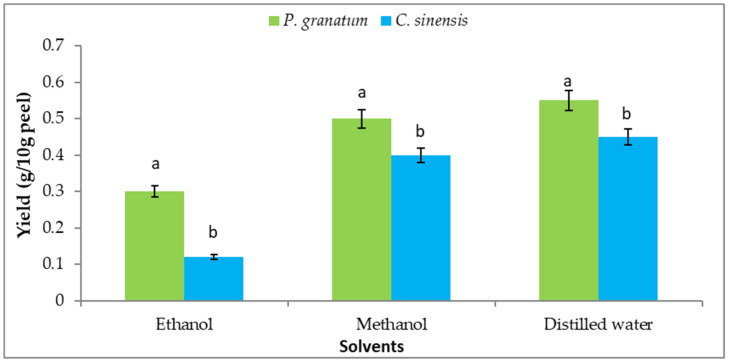
Yield of extracts from different peels utilizing various solvents. Each value is a mean (±SD) of three replicates. The different letters on the same bar show a significant difference according to Duncan’s test at *p* ≤ 0.05.

The selection of solvent is critical in order to obtain extracts with acceptable yields or high antioxidant activity. Water produced the highest yield (0.55 g/10 g peel), while an ethanol extract of pomegranate peel produced the lowest (0.3 g/10 g peel). In addition, the highest yield was detected in distilled water extract (0.45 g/10 g peel) and the lowest was detected in ethanolic extract of orange peel (0.12 g/10 g peel), as illustrated in Figure 7. Singh and Immanuel [105] extracted a yield of pomegranate and orange peel of about 27.5 and 23.9%; respectively.

### 3.9. Identification of Bacterial Isolates

**Figure 8:**
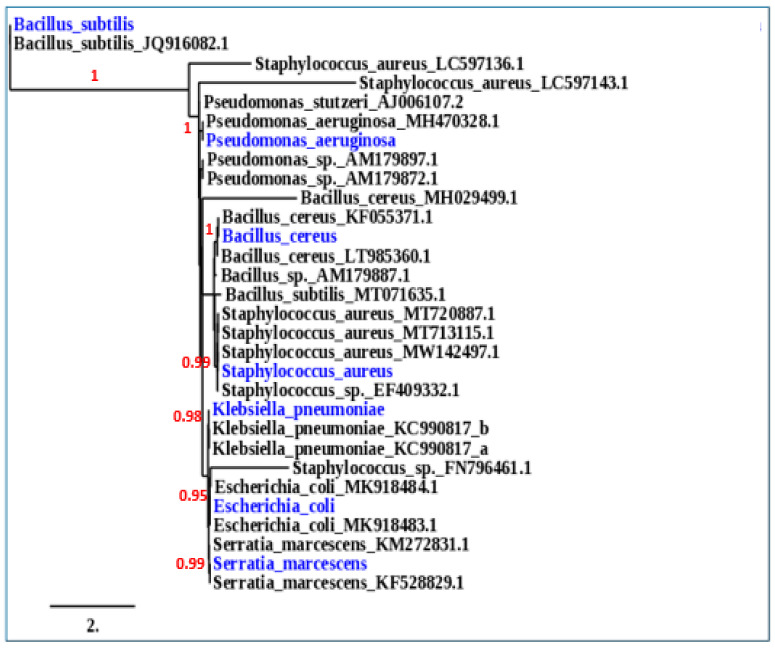
Phylogenetic trees of six bacterial isolates: E*scherichia coli*, *Pseudomonas aeruginosa*, *Klebsiella pneumonia*, *Staphylococcus aureus*, *Serratia marcesnces*, *Bacillus cereus*, and *Bacillus subtilis*.

Our seven isolates were identified phenotypically in Table 9 and genotypically in Figure 8. *E. coli*, *S. aureus*, *K. pneumonia*, *P. aeruginosa*, *S. marcesnces*, *B. cereus*, and *B. subtilis* were identified by using 16s rRNA sequences and comparing them with the Gen Bank database, with a similarity of 98–100%.

### 3.10. Antibacterial Activity of Fruits Peel Extracts

Herbs, trees, or bushes have been utilized by humans for decades in a variety of ways, including medications, foods, and flavors. The antibacterial activity of *C. sinensis* or *P. granatum* extracts was evaluated in vitro through agar disc and well diffusion against foodborne bacterial pathogens. Table 10 summarizes the microbial growth inhibition of all peel extracts. Compared to ethanolic extract, methanolic extract and distilled water had the highest antibacterial activity against foodborne bacteria. Ethanolic and methanolic extracts of *P. granatum* were effective against *K. pneumoniae* (19 mm, 25 mm), *P. aeruginosa* (19 mm, 27 mm), *S. marcescens* (23 mm, 28 mm), *S. aureus* (18 mm, 20 mm), or *E. coli* (20 mm, 21 mm), respectively. In addition, the ethanolic and methanolic extracts of *C. sinensis* also showed significant activity against *K. pneumoniae* (27 mm, 30 mm), *P. aeruginosa* (18 mm, 19 mm), *S. marcescens* (24 mm, 27 mm), *S. aureus* (22 mm, 26 mm), or *E. coli* (16 mm, 19 mm), respectively, whereas water extract had a high effect compared with the two used solvents. All bacterial pathogens were tested utilizing the activity index (AI) of extracts of amoxicillin antibiotics. Maximum inhibition was observed via *E. coli* (34 mm), *S. marcescens* (32 mm), and *P. aeruginosa* (32 mm) (30 mm).

The methanolic extract and distilled water of *P. granatum* peels had higher antibacterial activity against gram-negative bacteria like *P. aeruginosa*, *S. marcescens*, *E. coli*, or *K. pneumoniae* compared to *C. sinensis*. On the other hand, the methanolic extract and distilled water of *C. sinensis* showed higher antibacterial activity via *S. aureus*, *B. subtilis*, *B. cereus*, or *K. pneumoniae* compared to *P. granatum*. The antibacterial activity of *P. granatum* and *C. sinensis* peels could be attributed to the presence of metabolic toxins or broad-spectrum antimicrobial substances that act through gram +ve or gram −ve bacteria [86]. A variety of factors could impact the antibacterial activity of various fruit peel extracts. These criteria include the freshness of the used peels, the extraction process or solvent, the country in which the plant was grown, and the time of cultivation [106]. The presence of phenolic compounds have been linked to the antibacterial action of pomegranate and orange peel extracts [107]. In general, phenolic compounds produce antibacterial action via a variety of methods. These active chemicals can bind to the microbial cell wall and change its molecular structure or function, as well as denature several microbial enzymes. On the other hand, the existence of -OH groups in polyphenolic compounds may be related to the existence of the antibacterial effect by altering microorganism cell metabolism or the connection with vitamins, minerals, or carbohydrates, rendering them unattainable to microorganisms [108]. Pomegranate peel suppressed the growth of gram-positive *S. aureus* or Salmonella (gram-negative) [109].

According to the researchers, the use of various solvents to extract active compounds, as well as the concentration or temperature investigated, might change the level of active components in different species of fruit peels or thus, their antimicrobial capabilities [110]. Plant extracts, according to previous research by lvarezOrdóez et al. [111], can alter bacterial cell wall construction and cell membrane permeability, resulting in intracellular chemical leakage. According to certain research, gram-positive bacteria are much more vulnerable to plant extracts than gram-negative bacteria [112]. The outer cytoplasmic membrane encompassing the thin peptidoglycan structure of gram-negative bacteria that leads to restricted dispersion of hydrophobic compounds via their lipopolysaccharide covering may be the most significant reason for differences in microorganism vulnerability to antimicrobial agents [113]. Furthermore, the periplasmatic region includes enzymes capable of degrading foreign substances supplied from the outside. In gram-positive bacteria, antimicrobial agents can easily impact the cell wall or cytoplasmic membrane [113].

### 3.11. Minimum Inhibitory Concentration (MIC)

**Figure 9:**
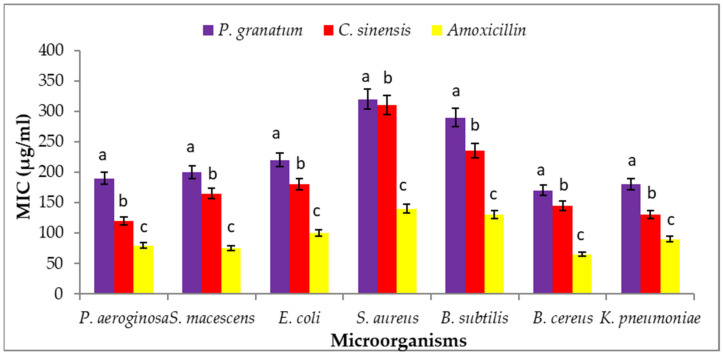
Antimicrobial activity of peel extracts by distilled water evaluated by minimum inhibitory concentration (MIC: µg/mL). Each value is a mean (±SD) of three replicates. The different letters on the same bar show a significant difference according to Duncan’s test at *p* ≤ 0.05.

The MIC concentration is reported in Figure 9. After treatment with *C. sinensis* and *P. granatum* peel, MIC data is presented for *P. aeruginosa* (190, 120 µg/mL), *S. macescens* (200, 165 µg/mL), *E. coli* (220, 180 µg/mL), *S. aureus* (320, 310 µg/mL), *B. subtilis* (290, 235 µg/mL) and *B. cereus* (170, 145 µg/mL).

The MIC results showed that a methanolic extract of peel could potentially act as a bactericidal agent against microorganisms. Karthikeyan and Vidya [114] showed that pomegranate peels have strong antibacterial action against *E. coli* or *B. subtilis*. Previous studies by Saleem et al. [115] also investigated the antibacterial potential of extracts of orange peel or yellow lemon peel. They observed that each citrus peel contained antimicrobial activity that was effective against a wide spectrum of bacteria. These scientists found MIC values for several orange peel extracts for *E. coli* or *S. aureus* in the ranges of 270–320 g/mL or 340–420 g/mL, respectively, that were greater than the quantity achieved in the current investigation [115]. Kharchoufi et al. [88] showed the antibacterial efficacy of pomegranate peel methanol extract against *Pseudomonas putida*. Malviya et al. [116] found that pomegranate peels have substantial antibacterial action via pathogenic bacteria strains like *Salmonella typhi*, *Enterobacter aerogenes*, or *S. aureus* in methanolic and aqueous extracts. Wafa et al. [117] determined that the MIC value of pomegranate peel ethanolic extract via Salmonella strains was in 10.75–12.50 mg/mL range.

### 3.12. Correlation Study

Results of correlational analysis are represented in Table 11. The findings indicate highly significant total flavonoid content (R = 0.998, *p* > 0.05) in cases of pomegranate peel extracts. However, no significant correlations were found between TFC or (R = −0.241). In the case of orange peel extract, a strong positive correlation (R = 0.997, *p* > 0.05) was noticed between the DPPH or scavenging capacity of the hydrogen peroxide. This might verify that substances that scavenge DPPH radicals in pomegranate peel extracts are also able to scavenge hydrogen peroxide.

### 3.13. Total Phenolic, Flavonoids, and Antioxidant Activity in Cakes after Addition of P. granatum and C. sinensis Peel Powder

**Figure 10:**
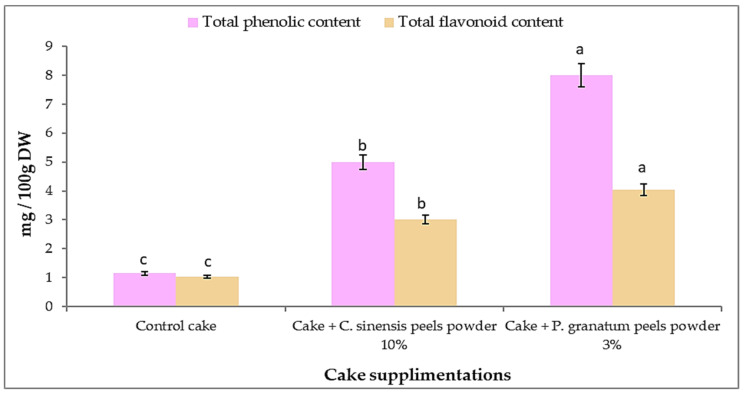
Total phenolic content (mg GAE/100 g DW) and total flavonoid content (mg QE/g DW) of cake supplemented with *C. sinensis* 10% and *P. granatum* 3% peel powder. Each value is a mean (±SD) of three replicates. The different letters on the same bar show a significant difference according to Duncan’s test at *p* ≤ 0.05.

**Figure 11:**
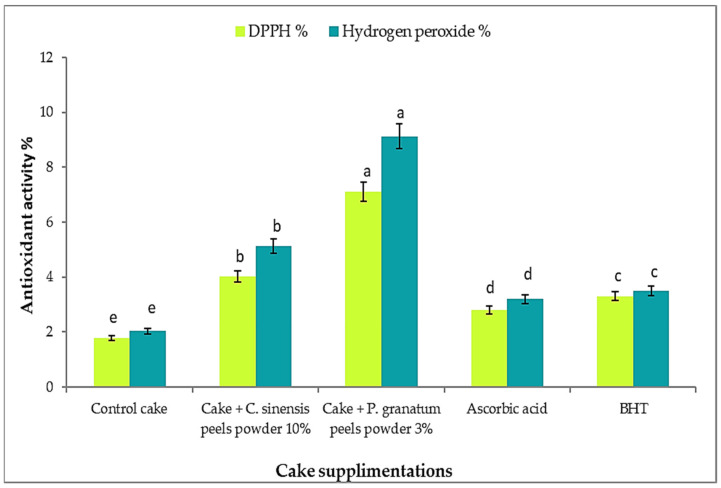
Antioxidant activity of cake supplemented with *C. sinensis* 10% and *P. granatum* 3% peel powder against DPPH^•^ and hydrogen peroxide. Each value is a mean (±SD) of three replicates. The different letters on the same bar show a significant difference according to Duncan’s test at *p* ≤ 0.05.

Phenolic compounds are known to contribute significantly to the sensory attributes of foods such as flavor, color, and taste. Furthermore, diets high in phenolic compounds are gaining popularity due to their bioactivity as antioxidants and anti-cancer agents. The increasing demand for phenolics in our diet has resulted in innovative strategies to supplement our diet with phenolic compounds in order to reap the health benefits [118]. According to preliminary studies, we added 10% orange peel powder and 3% pomegranate peel powder. Figure 10 and Figure 11 show that when pomegranate peel powder was added to cake samples, the percentage of total phenolic and flavonoid compounds increased when compared to the cake without any additions. When wheat flour was substituted with 3% pomegranate peel powder, the highest values of phenolic content (8.01 ± 0.2 mg GAE/g) and flavonoid content (4.02 ± 0.1 mg QE/g) were obtained, as compared to the control cake, which recorded 1.15 ± 0.1 mg GAE/g and 1.02 ± 0.01 mg QE/g, respectively. These findings are consistent with Lotfy and Barakat [119], who discovered that increasing the substituted ratios from zero to % pomegranate peel powder significantly increased the total phenolic content in the cake.

Furthermore, pomegranate peel powder incorporated into the cake demonstrated a significant (*p* ≤ 0.05) increase in and good capability for radical scavenging activity, ranging from 1.78% in the control cake to 7.11% in the pomegranate peel powder cake, and capability for hydrogen peroxide scavenging ranged from 2.03% in the control cake to 9.12% in the pomegranate peel powder cake. It is clear that these results are due to the high content of pomegranate peel powder in total phenolic compounds like flavonoids, polyphenols, and carotenes and antioxidant activity [120]. Therefore, it could be concluded that pomegranate peel powder can be considered a natural functional ingredient in bakery goods because of its high antioxidant properties.

These findings are consistent with those of Ismail et al. [121], who discovered that pomegranate phenolics’ ability to donate hydrogen atoms is one of the reported reasons for the high free radical scavenging characteristics of pomegranates and their extracts. There is a link between phenolic content and antioxidant activity. The presence of a significant concentration of phenolics in pomegranate-enriched cookies may play a disease-prevention role in addition to food storage. In addition, Prithwa and Sauryya [122] reported that enriching cookies with 2.5, 5, 7.5, and 10% pomegranate peel powder caused a significant increase in total phenolic content and antioxidant activity values. Also, Magda et al. [123] reported that the addition of orange peel powder to biscuit formulations has many advantages as an antioxidant to increase their shelf life and enhance the organoleptic properties of the biscuits. It can also reduce the amount of synthetic antioxidants.

### 3.14. The Effect of Shelf Life on Sensory Evaluation of Cake Supplemented with C. sinensis and P. granatum Peel Powder

**Figure 12:**
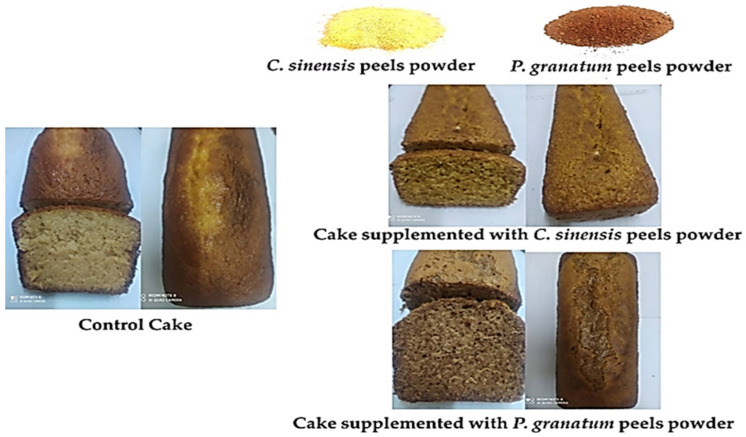
Photograph of cakes after baking (control cake, cake substituted with 3% pomegranate peel powder, and cake substituted with 10% orange peel powder).

The effect of shelf life on sensory evaluation of cake supplemented with pomegranate and orange peel powder at room temperature (30 °C) and refrigeration temperature (3 °C) is presented in Table 12 and Table 13 and Figure 12. The results showed that at room temperature, the taste and texture of pomegranate and orange peel powder differed from the control, whereas at refrigeration temperature, there were differences in all sensory evaluations compared to the control. This could be due to the length of time spent in the refrigerator. The addition of pomegranate and orange peel powder changed the taste and texture of the cake. This could be due to the extremely high fiber content of pomegranate and orange peel powder, which tends to make the cake rough. According to the findings, there was a difference in the overall acceptability between the cake supplemented with pomegranate and orange peel powder at room temperature and at refrigerator temperature. The overall acceptability of the cake samples varied according to storage time. The sensory evaluation of the supplemented cake remained nearly unchanged after 20 days in the refrigerator. However, the rejection threshold was only reached after 25 days.

The results show that pomegranate and orange peel powder have an effect on all sensory properties of the cake when compared to the control. These findings are consistent with those of Urganci and Isik [124], who recommended using pomegranate peel powder formulation rates of up to 18% in the formulation and discovered the possibility of using pomegranate peel powder in biscuits to enhance nutritional values. Furthermore, pomegranate peel powder will be accelerated as human feed, gaining more value as a waste product. Abd El-Galeel and Shoughy [125] discovered that incorporating 15% orange and mandarin peel powders in the formulation resulted in highly acceptable cakes. Furthermore, they revealed that citrus peel has a high content of essential oils, which incorporate some bitter compounds and give the product an accurate bitter taste when added at a higher concentration.

According to Lotfy and Barakat [119], the control sample outperformed the treated sponge cakes in all sensory attributes. After the control cake, the sponge cake containing 5% pomegranate peel powder had the second highest overall acceptability score (8.71), while sponge cake containing 20% pomegranate peel powder had the lowest (7.51). Sponge cakes with pomegranate peel powder substitution will be superior in sensory aspects. The taste score decreased significantly as the amount of pomegranate peel powder increased by 20%, which could be due to the slight bitterness of phenolic and tannin compounds. The phenolic compounds such as tannins that are found in the pomegranate peel powder sponge cakes could explain the observed color of the sponge cake [126,127,128]. Because the pomegranate peel powder had a dark brown color, the acceptability of the pomegranate peel powder sponge cake color was reduced with the addition of pomegranate peel powder. The taste score decreased slightly due to the high amount of pomegranate peel powder that was added, which was attributed to the presence of phenolic compounds with a bitter taste in the pomegranate peel powder [129].

Al-Saab and Gadallah [130] discovered that there was no significant difference in appearance when 5 and 10% wheat flour were substituted with orange peel powder in cookies, with values of 8.3 and 7.7, respectively, when compared to control cookies (8.1), while 20% orange peel powder resulted in the lowest value of appearance (7.2). Data also revealed that all levels of orange peel powder added had no negative effect on its color, whereas panelists accepted the cookie samples in terms of color up to 20% replacement. This is most likely due to the light yellow color of the peel powder, which enhances the color of the samples.

In terms of cookie texture, it is well known that the addition of fruit or vegetable peel powder results in a slight improvement in the hardness of the cookies. These findings are consistent with the findings of Zaker et al. [131], who noted that a slight increase in the crispiness of cookies was noticed in samples containing up to 10% peel powder, which secured higher scores; however, with 20% peel powder, the panelists indicated dryness of mouth, which secured the lowest scores. According to Haque et al. [132], as the orange fiber content increased, the texture of the cookies became harder and the acceptability level was reduced. In addition, Ismail et al. [121] discovered that the textural hardness feature of cookies has been linked to fiber content. A moderate and gradual increase in crude fiber content in pomegranate peel powder supplemented cookies may have a featured product hardening property, providing an additional characteristic sensorial score decrease.

Al-Saab and Gadallah [130] reported that there was no significant (*p* ≤ 0.05) difference in taste, until a substitution level up to 10% compared to the control (8.3), and an acceptance of the cookie sample containing 15% of orange peel powder was observed (7.2). The lowest taste value of 6.8 was recorded by cookies containing 20% orange peel powder. The bitterness and astringent taste were encountered in cookies as a result of the existence of alkaloids, tannins, and saponins in the orange fruit peel, as reported by Chikezie et al. [133]. Phenolic compounds are known to contribute immensely to sensory attributes such as flavor, color, and taste flavor of foods [118]. No significant (p ≤ 0.05) effect was observed in its aroma for all treatments with different ratios of orange peel powder as compared to the control sample.

Al-Saab and Gadallah [130] observed that there was no significant (*p* ≤ 0.05) difference in the overall acceptability between the control (8.4) and cookies incorporated with 5% orange peel powder (8.2). The samples were accepted by the panelists with up to 15% orange peel powder, as no negative effect was shown on the general acceptance of cookies. This data is in accordance with Mahmoud et al. [134] and Khule et al. [135]. Similarly, to the study conducted by Bourekoua et al. [136], it has been observed that the addition of pomegranate reduces the overall impression after consumption. This may be due to the slightly sour taste in general, which is dependent on the acids present in the pomegranate.

### 3.15. Analysis of Microbial Load in Cake

All cake samples were stored at room temperature (25 ± 1°C) and refrigerator (4°C) conditions for 17 days. Data in Table 14 and Table 15 showed that after 17 days of storage, a total microbial count of orange peel and pomegranate peel cakes were detected at room temperature and at refrigerator. However, total count for the control was detected after 2–6 days of storage at room temperature and at refrigerator conditions. Refer to Table 14 and Table 15 for mold detection after 4 days of control at room temperature and 6 days in the refrigerator. On the basis of these findings, it could be contended that the product is safe to consume due to proper hygienic considerations during preparation with peel cake. From our results, pomegranate peel is considered a better preservative than orange peel. Dimic et al. [137] indicated that the lemon essential oil showed a complete inhibition of the growth of the tested molds at ≥1.25 μL/mL using both types of contact tests, and it provided a natural system of food safety in both direct and vapor contact. The challenge is to maximize peel natural substances with biological activity and replace chemical additives [138,139,140].

## 4. Conclusions

The methanolic extract of *C. sinensis* or *P. granatum* peel powder was more effective than the other solvents. The aqueous and methanolic extracts had the highest yield values, i.e., flavonoid or phenolic content and chelating or antioxidant activities (%DPPH scavenging activity). In the GC−MS analysis of orange peel oil, 95.7% of limonene compound was detected. This is the major component of the orange peel oil and probably the source of the antibacterial and antifungal properties of the oils. The most potent antibacterial action was shown against *P. aeroginosa*, *K. pneumoniae*, *S. macescens*, and *E. coli*. Antibiotic resistance is quickly becoming a major issue. Our findings describe a novel antimicrobial extract that could be used instead of antibiotics to treat or prevent infectious bacterial illnesses. The use of pomegranate and orange peel powders in the manufacture of cake showed an increase in total phenolics, flavonoids, and antioxidant contents compared to control cake. The addition of pomegranate and orange peel powder to wheat flour was found to be promising in the shelf life extension of cookies by way of their high antioxidant potential. Furthermore, the utilization of pomegranate peel powder will help to decrease environmental pollution. The authors believe that pomegranate and orange peel powder can be used in other foods, especially in sweet baked goods as well as sweeter products such as jams and juices that can mask the bitter taste. More studies are needed to investigate other possible uses of pomegranate and orange peel powder. The obtained results could be commercialized by using the concentrated methanol extract of orange and pomegranate as a natural preservative (anti-bacterial) fragrance agent, especially from orange peels.

## Figures and Tables

**Table 1:** Raw ingredients of processed cakes according to Zaker et al. [65].

Ingredients	Weight (g)
Wheat flour (72% extraction)	250
Sugar	125
Milk powder	25
Fat	53.5
Fresh whole egg	110
Baking powder	12.5
Vanilla	2.0

**Table 2:** Physical observation of *P. granatum* and *C. sinensis* L. peel powder.

Peels	*P. granatum*	*C. sinensis* L.
Color	Dark brown color	Light brown color
Odor	Characteristic with tannin odor	Characteristic with pleasant odor
Appearance	Dark brown colored granular powder	Light brown colored granular powder

**Table 3:** Mineral composition of *P. granatum* and *C. sinensis* L. peels (mg/100 g DW).

Peels	Ca	Mg	Fe	Cu	P	K
*C. sinensis*	317 ± 5.0 ^b^	80.3 ± 3.0 ^b^	16.0 ± 0.8 ^b^	1.3 ± 0.1 ^b^	60.4 ± 3.0 ^a^	163.4 ± 6.0 ^a^
*P. granatum*	757.7 ± 7.0 ^a^	168.1 ± 4.0 ^a^	21.5 ± 0.9 ^a^	1.7 ± 0.2 ^a^	58.8 ± 4.0 ^b^	83.4 ± 5.0 ^b^

Values are means ± standard deviation (SD) of three replicates. The different letters on the same column show a significant difference according to Duncan’s test at *p* ≤ 0.05.

**Table 4:** Phytochemical screening of *C. sinensis* and *P. granatum* peel extracts.

Phytochemicals	*C. sinensis* Peel			*P. granatum* Peel		
	Ethanol	Methanol	Dist. Water	Ethanol	Methanol	Dist. Water
Alkaloids	+	++	++	++	+++	+++
Tannins	+	++	++	++	+++	+++
Cardiac glycosides	+	+	+	+	+	+
Flavonoids	++	++	++	++	+++	+++
Glycosides	+	++	++	+	−	−
Phenols	+	++	++	++	+++	+++
Saponins	+	++	−	++	++	−
Steroids	+	++	++	++	+++	+++
Terpenoids	+	++	++	++	++	+++

The symbols +++, ++, +, and − denote appreciable amounts (positive within 5 min); moderate amounts (positive after 5 min but within 10 min); trace amounts (positive after 10 min but within 15 min) and completely absent, respectively.

**Table 5:** Total phenolic and total flavonoid of *C. sinensis* and *P. granatum* peel extract.

Peel Powder	*C. sinensis*		*P. granatum*	
Extract	Total Phenolic Content mg GAE/100 g DW	Total Flavonoid Content mg QE/g DW	Total Phenolic Content mg GAE/100 g DW	Total Flavonoid Content mg QE/g DW
Ethanol	143.7 ± 2.0 ^c^	10.2 ± 0.3 ^c^	350.4 ± 3.0 ^c^	20.5 ± 0.2 ^c^
Methanol	155.4 ± 2.0 ^b^	15.7 ± 0.3 ^b^	490.6 ± 4.0 ^b^	40.3 ± 0.3 ^b^
Dist. Water	160.3 ± 3.0 ^a^	22.2 ± 0.5 ^a^	513.8 ± 4.0 ^a^	45.3 ± 0.5 ^a^

Values are means ± standard deviation (SD) of three replicates. The different letters on the same column show a significant difference according to Duncan’s test at *p* ≤ 0.05.

**Table 6:** Phenolic compounds in the *C. sinensis* peels by LC-MS/MS at negative ion mode.

Compounds Class	Phenolic Compounds	Base Peak *m*/*z*	[M-H]^−^	Retention Time (min)	Relative Percentage
Flavonoid	Naringin	271	579	14.189	31.75
Flavonoid	Vicenin II	299	593	15.802	6.61
Flavonoid	Eriodictyol-7-rutinoside	449	595	16.11	1.63
Flavonoid	Apigenin 7-O-neohesperidoside (Rhoifolin)	269	577	17.229	5.91
Flavonoid	Hesperidin	301	609	17.326	10.13
Flavonoid	Eriocitrin	289	595	17.587	1.96
Flavonoid	Naringenin	271	271	17.625	1.31
Flavonoid	Neohesperidin	301	609	18.793	3.9
Flavonoid	Luteolin-7-O-rutinoside	271	593	21.708	0.7
Flavonoid	Sinensetin	371	371	21.956	0.98
Flavonoid	Nobiletin	387	401	23.175	1.42
Flavonoid	3-methoxynobiletin	417	431	23.458	0.96
Flavonoid	Tangeritin	353	371	24.994	21.11

**Table 7:** Phenolic compounds in the *P. granatum* peels by LC-MS/MS.

Compounds Class	Phenolic Compounds	Base Peak *m*/*z*	[M-H]^−^	Retention Time (min)	Relative Percentage
Phenolic acid	*p*-cumaric	164	164	24.324	0.74
Tanines	Gallic	170	170	25.634	17.44
Phenolic acid	Syringic	198	198	25.709	0.43
Phenolic acid	Caffeic	180	180	25.944	2.05
Tannins	Alpha-punicalagin	782	1084	26.127	6.34
Tannins	Beta-punicalagin	782	1084	27.106	9.25
Flavonoid	Pelargonidin	271	271	27.646	1.41
Flavonoid	Epicatchin	139	290	28.277	2.35
Tannins	Punicalin	601	782	28.521	0.96
Flavonoid	Catchin	290	290	29.275	2.67
Phenolic acid	Ellagic	302	302	30.12	16.54
Flavonoid	Quercetin	302	302	31.191	5.64
Phenolic acid	Chlorogenic	191	354	32.175	2.3
Flavonoid	Cyanidin-3-O-glucoside	317	484	34.757	14.54
Flavonoid	Pelargonidin-3-glucoside	359	433	34.922	5.44
Flavonoid	Cyanidin-3,5-diglucoside	301	611	36.654	2.04
Flavonoid	Rutin	580	610	36.782	1.72
Flavonoid	Delphinidin 3,5-diglucoside	527	627	37.898	1.94
Tannins	Pedunculagin	782	1084	43.245	0.71
Tannins	Granatin A	784	951	43.375	0.24

**Table 8:** Essential oil compounds in *C. sinensis* peels by GC-MS.

Essential Oil Compounds	Retention Time (min)	Relative Percentage
β-Myrcene	5.418	1.42
D-Limonene	6.254	95.7
β-Linalool	7.823	1.73
Decanal	10.393	0.58
Valencene	17.815	0.57

**Table 9:** Morphological and biochemical tests of six bacterial isolates.

Microorganisms	Gram Stain	Motility	Spore Formation	Shape	Catalase	Oxidase	Nitrate Reductase	Gelatine Hydrolysis	Indole	MR	VP	Urease
*Pseudomonas* *aeroginosa*	−	+	−	Short rod	+	+	+	+	−	−	−	−
*Serratia macescens*	−	+	−	Short rod	+	−	+	+	−	−	+	+
*E. coli*	−	+	−	Short rod	+	−	+	−	+	+	−	−
*Staphylococcus aureus*	+	−	−	Cocci	+	−	+	+	−	+	+	+
*Bacillus subtilis*	+	+	+	Rod	+	−	+	+	−	−	+	−
*Bacillus cereus*	+	+	+	Rod	+	−	−	−	−	−	+	−
*Klebsiella pneumonia*	−	−	−	Short rod	+	−	+	−	−	+	+	+

**Table 10:** Antimicrobial activity of peel extracts (ethanolic, methanolic, and distilled water) against different microorganisms.

Zone of Inhibition (mm)							
Peel Extract	*P. granatum*			*C. sinensis*			Antibiotic
Solvent	Eth.	Meth.	DW	Eth.	Meth.	DW	Amoxicillin
Microorganism							
*P. aeruginosa* (−ve)	19 ± 0.1 ^d^	27 ± 0.3 ^b^	30 ± 0.3 ^a^	18 ± 0.1 ^e^	19 ± 0.1 ^d^	21 ± 0.1 ^c^	30 ± 0.5 ^a^
*S. marcescens* (−ve)	23 ± 0.2 ^f^	28 ± 0.3 ^c^	33 ± 0.3 ^a^	24 ± 0.2 ^e^	27 ± 0.3 ^d^	27 ± 0.2 ^d^	32 ± 0.5 ^b^
*E. coli* (−ve)	20 ± 0.2 ^d^	21 ± 0.2 ^c^	25 ± 0.2 ^b^	16 ± 0.1 ^f^	19 ± 0.1 ^e^	21 ± 0.1 ^c^	34 ± 0.4 ^a^
*S. aureus* (+ve)	18 ± 0.1 ^f^	20 ± 0.2 ^e^	22 ± 0.1 ^d^	22 ± 0.2 ^d^	26 ± 0.2 ^c^	32 ± 0.3 ^a^	29 ± 0.4 ^b^
*B. subtilis* (+ve)	17 ± 0.1 ^f^	18 ± 0.1 ^e^	20 ± 0.1 ^c^	18 ± 0.1 ^e^	19 ± 0.1 ^d^	23 ± 0.1 ^b^	28 ± 0.4 ^a^
*B. cereus* (+ve)	18 ± 0.1 ^e^	19 ± 0.1 ^d^	22 ± 0.1 ^b^	15 ± 0.1 ^f^	21 ± 0.1 ^c^	24 ± 0.1 ^a^	22 ± 0.3 ^b^
*K. pneumoniae* (−ve)	19 ± 0.1 ^f^	25 ± 0.2 ^e^	28 ± 0.2 ^c^	18 ± 0.1 ^g^	27 ± 0.2 ^d^	30 ± 0.3 ^a^	29 ± 0.4 ^b^

Each value is a mean (±SD) of three replicates. The different letters on the same column show a significant difference according to Duncan’s test at *p* ≤ 0.05.

**Table 11:** Correlation matrix between polyphenol composition or antioxidant activity of orange and pomegranate peel.

	TPC (mg GAE/100 g DW)	TFC (mg QE/g DW)	DPPH %	H_2_O_2_%
Orange peel				
TPC (mg GAE/100 g DW)	1	−0.241	−0.647	−0.796
TFC (mgQE/g DW)	−0.241	1	0.896	0.779
DPPH %	−0.647	0.896	1	0.977
H_2_O_2_	−0.796	0.779	0.977	1
Pomegranate peel				
TPC (mg GAE/100 g DW)	1	0.998	−0.259	−0.171
TFC (mgQE/g DW)	0.998	1	−0.317	−0.111
DPPH %	−0.259	−0.317	1	−0.908
H_2_O_2_	−0.171	−0.111	−0.908	1

**Table 12:** Effect of shelf life on sensory evaluation for cake supplemented with *C. sinensis* and *P. granatum* peel powder at room temperature (30 °C).

Samples	Storage Time (Days)	Color	Appearance	Taste	Texture	Aroma	Overall Acceptability
Control cake	Day 0	8.19 ± 0.26 ^a^	8.57 ± 0.12 ^a^	8.35 ± 0.27 ^a^	8.62 ± 0.23 ^a^	8.90 ± 0.31 ^a^	8.03 ± 0.45 ^a^
	Day 5	7.85 ± 0.31 ^b^	6.98 ± 0.24 ^d^	not edible	6.02 ± 0.30 ^e^	6.43 ± 0.27 ^d^	6.05 ± 0.31 ^e^
	Day 10	6.30 ± 0.13 ^e^	5.62 ± 0.11 ^h^	not edible	4.31 ± 0.13 ^g^	4.69 ± 0.21 ^f^	3.46 ± 0.11 ^g^
*C. sinensis* peels fortified cake 10%	Day 0	6.01 ± 0.26 ^f^	6.01 ± 0.16 ^g^	6.69 ± 0.34 ^e^	6.98 ± 0.28 ^d^	8.03 ± 0.22 ^b^	6.99 ± 0.16 ^d^
	Day 5	7.01 ± 0.29 ^d^	7.14 ± 0.12 ^c^	7.01 ± 0.10 ^d^	7.03 ± 0.29 ^d^	7.57 ± 0.37 ^c^	6.89 ± 0.15 ^d^
	Day 10	6.02 ± 0.21 ^f^	5.42 ± 0.18 ^i^	not edible	4.05 ± 0.17 ^h^	4.27 ± 0.22 ^g^	4.01 ± 0.13 ^f^
*P. granatum* peel-fortified cake 3%	Day 0	6.12 ± 0.19 ^f^	6.67 ± 0.17 ^e^	7.03 ± 0.15 ^c^	7.53 ± 0.28 ^c^	8.92 ± 0.47 ^a^	7.17 ± 0.10 ^c^
	Day 5	8.09 ± 0.20 ^a^	8.43 ± 0.11 ^b^	7.89 ± 0.21 ^b^	8.03 ± 0.20 ^b^	8.01 ± 0.19 ^b^	7.69 ± 0.34 ^b^
	Day 10	7.53 ± 0.38 ^c^	6.24 ± 0.11 ^f^	not edible	5.34 ± 0.24 ^f^	5.63 ± 0.26 ^e^	4.15 ± 0.14 ^f^

Each value is a mean (±SD) of three replicates. The different letters on the same bar show a significant difference according to Duncan’s test at *p* ≤ 0.05.

**Table 13:** Effect of shelf life on sensory evaluation for cake supplemented with *C. sinensis* and *P. granatum* peel powder at refrigeration temperature (3 °C).

Samples	Storage Time (Days)	Color	Appearance	Taste	Texture	Aroma	Overall Acceptability
Control cake	Day 0	8.19 ± 0.14 ^a^	8.57 ± 0.13 ^a^	8.35 ± 0.18 ^a^	8.62 ± 015 ^a^	8.90 ± 0.31 ^a^	8.03 ± 0.17 ^a^
	Day 5	8.18 ± 0.18 ^a^	8.50 ± 0.14 ^a^	8.01 ± 0.11 ^b^	7.54 ± 0.18 ^b^	8.01 ± 0.33 ^c^	7.53 ± 0.16 ^b^
	Day 10	8.01 ± 0.15 ^b^	8.43 ± 0.16 ^a^	7.04 ± 0.17 ^c^	6.43 ± 0.16 ^f^	7.68 ± 0.11 ^e^	6.54 ± 0.21 ^e^
	Day 15	7.52 ± 0.19 ^c^	7.89 ± 0.24 ^b^	6.31 ± 0.29 ^e^	5.37 ± 0.11 ^i^	6.89 ± 0.23 ^g^	6.01 ± 0.21 ^f^
	Day 20	6.63 ± 0.30 ^d^	6.55 ± 0.14 ^c^	not edible	4.99 ± 0.18 ^j^	5.87 ± 0.14 ^j^	5.03 ± 0.34 ^i^
	Day 25	6.57 ± 0.21 ^d^	5.45 ± 0.18 ^h^	not edible	4.01 ± 0.11 ^l^	5.01 ± 0.15 ^m^	4.02 ± 0.18 ^k^
*C. sinensis* peel-fortified cake 10%	Day 0	6.01 ± 0.26 ^e^	6.01 ± 0.17 ^f^	6.69 ± 0.16 ^d^	6.98 ± 0.19 ^d^	8.03 ± 0.26 ^c^	6.99 ± 0.20 ^d^
	Day 5	6.00 ± 0.13 ^e^	6.00 ± 0.14 ^f^	6.34 ± 0.14 ^e^	6.64 ± 0.14 ^e^	8.00 ± 0.28 ^c^	6.57 ± 0.17 ^e^
	Day 10	5.34 ± 0.19 ^g^	5.78 ± 0.18 ^g^	6.03 ± 0.08 ^f^	6.03 ± 0.13 ^g^	7.45 ± 0.20 ^f^	6.02 ± 0.23 ^f^
	Day 15	5.22 ± 0.08 ^g^	5.26 ± 0.13 ^i^	5.41 ± 0.18 ^g^	5.73 ± 0.17 ^h^	7.04 ± 0.18 ^g^	5.47 ± 0.10 ^g^
	Day 20	5.11 ± 0.11 ^h^	5.02 ± 0.12 ^j^	4.23 ± 0.05 ^i^	5.35 ± 0.06 ^i^	6.09 ± 0.12 ^i^	5.02 ± 0.10 ^i^
	Day 25	5.01 ± 0.09 ^h^	4.89 ± 0.11 ^k^	not edible	4.89 ± 0.08 ^k^	5.34 ± 0.11 ^l^	4.65 ± 0.13 ^j^
*P. granatum* peel-fortified cake 3%	Day 0	6.12 ± 0.17 ^e^	6.67 ± 0.21 ^c^	7.03 ± 0.12 ^c^	7.53 ± 0.13 ^b^	8.92 ± 0.44 ^a^	7.17 ± 0.14 ^c^
	Day 5	6.02 ± 0.20 ^e^	6.57 ± 0.17 ^c^	7.00 ± 0.11 ^c^	7.34 ± 0.17 ^c^	8.46 ± 0.42 ^b^	7.02 ± 0.11 ^d^
	Day 10	6.00 ± 0.22 ^e^	6.50 ± 0.11 ^c^	6.98 ± 0.15 ^c^	7.01 ± 0.18 ^d^	7.87 ± 0.11 ^d^	6.89 ± 0.14 ^d^
	Day 15	5.93 ± 0.14 ^e^	6.43 ± 0.10 ^d^	6.01 ± 0.14 ^f^	6.45 ± 0.19 ^f^	7.30 ± 0.14 ^f^	6.10 ± 0.16 ^f^
	Day 20	5.78 ± 0.10 ^f^	6.32 ± 0.08 ^e^	4.69 ± 0.09 ^h^	5.69 ± 0.16 ^h^	6.34 ± 0.19 ^h^	5.34 ± 0.11 ^h^
	Day 25	5.74 ± 0.18 ^f^	6.31 ± 0.09 ^e^	not edible	5.01 ± 0.14 ^j^	5.68 ± 0.15 ^k^	5.03 ± 0.24 ^i^

Each value is a mean (±SD) of three replicates. The different letters on the same bar show a significant difference according to Duncan’s test at *p* ≤ 0.05.

**Table 14:** Antimicrobial effect of orange and pomegranate peel on storage cake samples at refrigerator temperatures (cfu/g).

Treatments	Zero Time		2 Days		4 Days		6 Days		13 Days		15 Days		17 Days	
	Total Count	Mold Count	Total Count	Mold Count	Total Count	Mold Count	Total Count	Mold Count	Total Count	Mold Count	Total Count	Mold Count	Total Count	Mold Count
Control	0	0	0	0	0	0	4 × 10^2^	3 × 10^2^	ND	ND	ND	ND	ND	ND
Orange peel	0	0	0	0	0	0	0	0	0	0	3 × 10^2^	2 × 10^2^	4 × 10^2^	2 × 10^2^
Pomegranate peel	0	0	0	0	0	0	0	0	0	0	2 × 10^2^	2 × 10^2^	3 × 10^2^	2 × 10^2^

**Table 15:** Antimicrobial effect of orange and pomegranate peel on storage cake samples at room temperature (cfu/g).

Treatments	Zero Time		2 Days		4 Days		6 Days		13 Days		15 Days		17 Days	
	Total Count	Mold Count	Total Count	Mold Count	Total Count	Mold Count	Total Count	Mold Count	Total Count	Mold Count	Total Count	Mold Count	Total Count	Mold Count
Control	0	0	0	0	6 × 10^2^	3 × 10^2^	11 × 10^2^	7 × 10^2^	ND	ND	ND	ND	ND	ND
Orange peel	ND	ND	ND	ND	ND	ND	ND	ND	4 × 10^2^	2 × 10^2^	7 × 10^2^	5 × 10^2^	11 × 10^2^	8 × 10^2^
Pomegranate peel	ND	ND	ND	ND	ND	ND	ND	ND	3 × 10^2^	1 × 10^2^	5 × 10^2^	3 × 10^2^	9 × 10^2^	6 × 10^2^

## Data Availability

16S rRNA sequences of isolated bacterial strains were submitted to the NCBI database.
